# Integrative Taxonomy and Molecular Phylogeny of Genus *Aplysina* (Demospongiae: Verongida) from Mexican Pacific

**DOI:** 10.1371/journal.pone.0042049

**Published:** 2012-08-13

**Authors:** José Antonio Cruz-Barraza, José Luis Carballo, Axayacatl Rocha-Olivares, Hermann Ehrlich, Martin Hog

**Affiliations:** 1 Unidad Académica Mazatlán, Instituto de Ciencias del Mar y Limnología, Universidad Nacional Autónoma de México (UNAM), Mazatlán Sinaloa, México; 2 Departamento de Oceanografía Biológica, Centro de Investigación Científica y de Educación Superior de Ensenada (CICESE), Ensenada, Baja California, México; 3 Institute of Bioanalytical Chemistry, Dresden University of Technology, Dresden, Germany; University of Veterinary Medicine Hanover, Germany

## Abstract

Integrative taxonomy provides a major approximation to species delimitation based on integration of different perspectives (e.g. morphology, biochemistry and DNA sequences). The aim of this study was to assess the relationships and boundaries among Eastern Pacific *Aplysina* species using morphological, biochemical and molecular data. For this, a collection of sponges of the genus *Aplysina* from the Mexican Pacific was studied on the basis of their morphological, chemical (chitin composition), and molecular markers (mitochondrial COI and nuclear ribosomal rDNA: ITS1-5.8-ITS2). Three morphological species were identified, two of which are new to science. *A. clathrata* sp. nov. is a yellow to yellow-reddish or -brownish sponge, characterized by external clathrate-like morphology; *A. revillagigedi* sp. nov. is a lemon yellow to green, cushion-shaped sometimes lobate sponge, characterized by conspicuous oscules, which are slightly elevated and usually linearly distributed on rims; and *A. gerardogreeni* a known species distributed along the Mexican Pacific coast. Chitin was identified as the main structural component within skeletons of the three species using FTIR, confirming that it is shared among Verongida sponges. Morphological differences were confirmed by DNA sequences from nuclear ITS1-5.8-ITS2. Mitochondrial COI sequences showed extremely low but diagnostic variability for *Aplysina revillagigedi* sp. nov., thus our results corroborate that COI has limited power for DNA-barcoding of sponges and should be complemented with other markers (e.g. rDNA). Phylogenetic analyses of *Aplysina* sequences from the Eastern Pacific and Caribbean, resolved two allopatric and reciprocally monophyletic groups for each region. Eastern Pacific species were grouped in general accordance with the taxonomic hypothesis based on morphological characters. An identification key of Eastern Pacific *Aplysina* species is presented. Our results constitute one of the first approximations to integrative taxonomy, phylogeny and evolutionary biogeography of Eastern Pacific marine sponges; an approach that will significantly contribute to our better understanding of their diversity and evolutionary history.

## Introduction

Taxonomy and species recognition are a fundamental basis for all theoretical and applied biological research. For centuries, traditional taxonomy has been based on comparative morphology, and even today most species descriptions are mainly based on morphology. However, this traditional approach can bear some subjectivity in the interpretation of characters, making the identification of species difficult, and causing unstable systematics across entire taxa (e. g. Porifera) [1], [2]. Technological advances have provided new tools that facilitate obtaining different kinds of biological data (e. g. micro morphology, biochemistry, ecology, genetics etc.), which are confronted in taxonomy and evolutionary studies of the species [3]. DNA barcoding [4] has become a particularly efficient tool in the identification and delimitation of new and known species from various groups [5]–[7]. Although it has demonstrated limited resolution power in some cases [8]–[10].

In the last decade, an “integrative taxonomy” combining multiple kinds of data and complementary perspectives (i.e., phylogeography, comparative morphology, population genetics, ecology, development, behavior, etc.) has been recognized as the most objective means to delimit the units of life's diversity (see [3], [11]). Several operational approaches have emerged to implement an integrative taxonomy, such as the “taxonomic circle”, among others [3], [12]. The taxonomic circle seeks to reconcile different types of characters in order to determine species boundaries through a process of hypothesis testing, corroboration, reciprocal illumination and revision. These approaches have been well received and adopted in several recent studies, increasing the use of combined lines of evidence to test taxonomic hypothesis of species status [3], [12], [13].

The Phylum Porifera is a very diverse and abundant group in the marine benthic ecosystem [14]. Currently, sponges have attracted a growing interest due to their evolutionary, ecological and economical importance (particularly in the pharmaceutical, biomaterial and biotechnology fields) (e. g. [14]–[16]). However, their taxonomic complexity makes species identification difficult and consequently hinders their potential application. The problem increases in groups lacking a mineral skeleton (e.g. horny sponges), where morphological characteristics hardly vary, making it difficult to establish the boundaries between species and challenging their phylogenetic interpretation. However, the combined use of several markers such as morphology and DNA sequences has generated major comprehensive information about species determination, this is particularly relevant when morphological characters used for taxonomic interpretation are unstable (e. g. [17], [18]) and there is no molecular standard fragment for species discrimination.

Horny sponges (Demospongiae; orders Verongida, Dictyoceratida, and Dendroceratida) have a skeleton formed by spongin, a protein resulting from a super-compaction of collagen fibrils and filaments [19], [20]. However, phylogenetic and embryological studies have shown that Verongida, Dictyoceratida, and Dendroceratida – although characterized by fibrous spongin skeletons – do not make up a cohesive phylogenetic unit [21], [22]. Recent re-examination showed that chitin, rather than spongin, forms the main organic component of verongid skeletal fibres [23]. The presence of chitinous skeletons in different representatives of Verongida sponges [23]–[27] seems to be a characteristic property of this group, which has apparently evolved independently from Dictyoceratida and Dendroceratida [21]. Because sponges are often regarded as the most ancient metazoans, the finding of chitin in their skeleton is of major evolutionary significance.

Species of *Aplysina* (Order Verongida) are common inhabitants of shallow tropical and subtropical marine waters [28], [29]. They are usually large sponges, variable in form (massive, tubular, ramose, pedunculate, etc.), and with live colors, usually ranging from yellow to green [28], [30]. The genus is characterized by having a skeleton of pithed fibers in a single category, forming a regular reticulum of polygonal meshes without an ectosomal specialized structure [29]. In contrast to other keratose sponges, *Aplysina* skeletons are mostly made not of spongin, but of alpha-chitin [23]–[25], which gives this group an interesting biomedical potential in tissue engineering [24], [25].

*Aplysina*-species are also recognized by the possession of brominated alkaloid compounds with cytotoxic activities [31], [32], and microbial symbionts producing compounds with antibiotic activity [33].

The systematic history of the group is quite complex [29], [34]–[36]. The name *Aplysina* was established by Nardo (1834) and was commonly used, during the early 19^th^ and 20^th^ centuries, although some authors (e.g. [37]–[39]) used *Verongia* Bowerbank (1845). De Laubenfels (1948) presented an extensive discussion in order to establish *Verongia* instead of *Aplysina*. He proposed to synonymize *Aplysina* with *Spongia*, and to establish *Verongia* as the valid name for species described as *Aplysina*. Finally, Wiedenmayer (1977) resolved the status of the name *Aplysina* in relation to *Verongia* under the international code of zoological nomenclature, stabilizing the validity of genus *Aplysina* as a senior name over *Verongia* (see [35]).

The classification of *Aplysina* was first based on external morphology and skeleton, and was later complemented with reproductive, histological and biochemical characteristics [29], [34]. However, the absence of a mineral skeleton has limited species identification and the analysis of phylogenetic relationships in the group, particularly in the face of potential cryptic species and extreme phenotypic plasticity, which remain largely unapprised in these sponges.

Molecular markers have been increasingly used to resolve sponge relationships [2], [40]. DNA sequences from the mitochondrial (mtDNA) gene coding for the cytochrome oxidase subunit 1 (COI) have shown low resolution for the discrimination of *Aplysina* species [41], [42]. Partial sequences of the nuclear ribosomal DNA (rDNA), which may include the 18S, ITS1, 5.8S, ITS2, and 28S genes, have been successfully used to infer phylogenies from the ordinal- [42], [43] to species-level in Verongida [29]. More recently, analyses of single-stand conformational polymorphisms (SSCP) of PCR-amplified ITS have also been used to elucidate speciation patterns of *Aplysina* species, showing concordance between morphological and genetic data for species discrimination [44].

To date, only two valid species of genus are known in the Eastern Pacific: *Aplysina gerardogreeni* Gómez and Bakus 1992 (from Mexico) and *A. chiriquensis* Diaz et al. 2005 (from Panama to Galapagos Islands). Even though other congeneric species have been cited, most of them may be invalid due to the absence of detailed morphological descriptions (see discussion).

Here we analyze the status of the genus *Aplysina* in the Eastern Pacific using an integrative inferential approach based on morphological, chemical and molecular data to establish species boundaries and to unravel their evolutionary patterns. We describe two new species as well as new material of *A. gerardogreeni* based on morphological and molecular data. We also analyze the phylogenetic relationships of the Mexican *Aplysina* with other congeneric species from the Caribbean. Finally, a morphological identification key is provided for the species of *Aplysina* from the Eastern Pacific.

## Results

### Systematic descriptions

Order **Verongida** Bergquist, 1978

Family **Aplysinidae** Carter, 1875

Genus ***Aplysina*** Nardo, 1834

#### Diagnosis

Aplysinidae characterized by possession of fibers of only one kind with no foreign detritus and having a thick pith component. The fibers form a regular reticulum with large polygonal meshes and no specialized surface arrangement [29].

**Figure 1:**
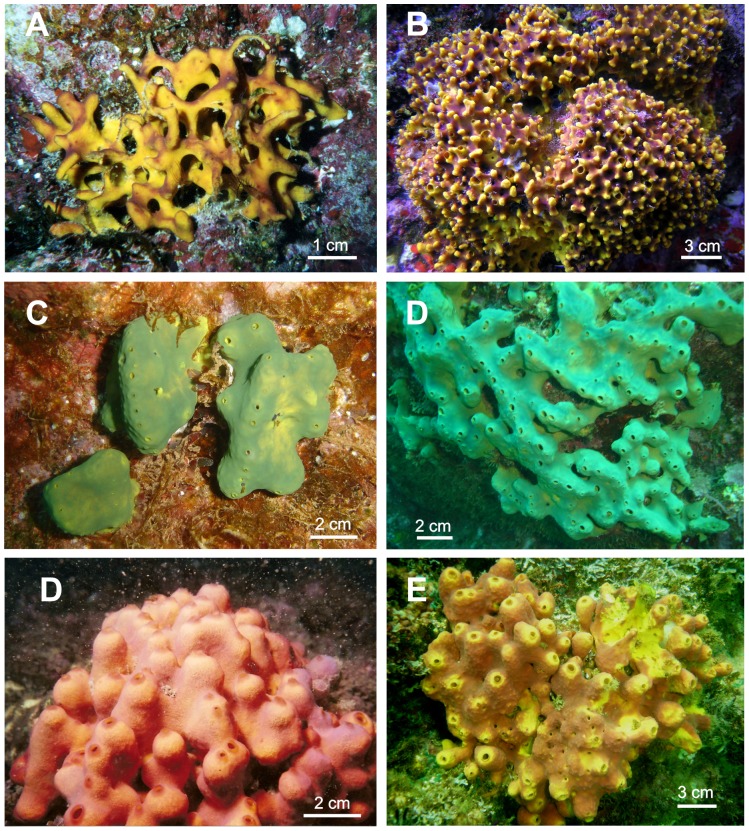
External morphology of Mexican Pacific *Aplysina* species. A,B) *Aplysina clathrata* sp. nov.; C,D) *Aplysina revillagigedi* sp. nov.; E,F) *Aplysina gerardogreeni*.

**Figure 2:**
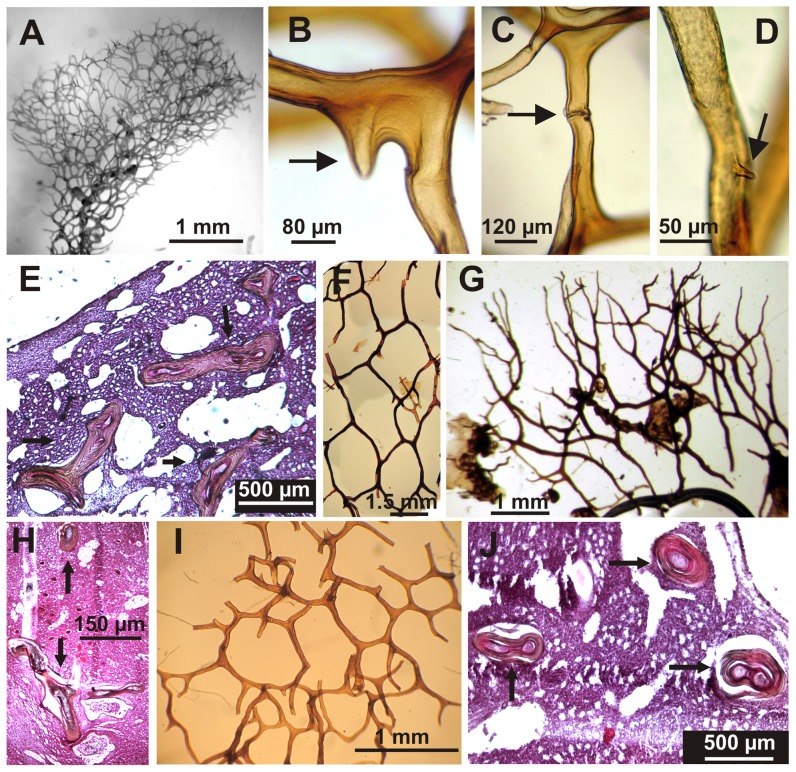
Skeletal characteristics of Mexican Pacific *Aplysina* species. A–E) *A. clathrata* sp. nov.; A) Regular tridimensional skeletal reticulation of a fistular proyection; B,C,D) Detail of skeleton sponging fibers with nodular pith and short protuberances (showing by arrows); E) Transversal view of fibers showing by arrows; F–H) *A. revillagigedi* sp. nov.; F) Tridimensional skeletal reticulation at deep choanosome; G) Dentritic-like terminal skeletal fibers; H) Transversal view of fibers showing by arrows; I,J) *A. gerardogreeni*; I) Regular tridimentional skeletal reticulation; J) Transversal view of fibers showing by arrows.

***Aplysina clathrata*** **sp. nov.**

(Figs. 1 A, B; 2 A–E)

#### Material examined

Holotype: MNCN 1.01/637, Cerro Pelón, isla Isabel (Nayarit) 21°51′21″N, 105°53′33″W, 15 m depth, 01/30/2003. Paratypes: BMNH 2010.11.01.7, Cerro Pelón, isla Isabel (Nayarit) 21°51′21″N, 105°53′33″W, 18 m depth, 01/27/2003. LEB-ICML-UNAM-57, bahía Tiburones, isla Isabel (Nayarit), 21°50′33″N, 105°53′10″W, 12 m depth, 11/20/1999. LEB-ICML-UNAM-2072, Los Islotes, Isla Espíritu Santo (Baja California Sur), 24°35′57″N, 110°24′04″W, 6 m depth, 08/10/2009.

GenBank accessions rDNA: JN596957 and : JN596956; mDNA-COI: JQ437579.

The ZooBank LSID: urn:lsid:zoobank.org:act:0BB11475-F8AD-4188-BC96-FEED8B09D865.

#### Description

Semi-spherical to massive reticulated or clathrate sponge, up to 40 cm in diameter, and from 3 to 10 cm high. The branches are from 2 to 8 mm in thickness, which form meshes from 0.3 to 1.5 cm in opening. Reticulum is more open in young specimens and becomes narrow in larger specimens (Fig. 1 A, B). Surface with lobules rounded, from 3 to 9 mm high and from 2 to 5 mm in diameter, slightly widened in the distal part. Occasionally they are fused forming lobes up to 1.5 cm long. The surface of the lobules is smooth, sometimes minute-conulose due to the tip of the fibers. Conula (from 100 to 300 µm) are more evident in preserved specimens. Ostial apertures are from 15 to 85 µm in diameter, and they are regularly distributed on surface. Oscules are relatively large, conspicuous, circular to oval-shaped, 2–4 mm in diameter, and distributed regularly around the surface, mainly between lobes. They are surrounded by a lightly elevated diaphragm-like membrane without skeletal fibers. The consistency is soft and flexible, harder in preserved specimens. Ectosome is a thin layer 66 to 117 µm thick, easily detachable in some parts of the body. The choanosome is cavernous, with circular to oval shaped channels from 40 a 160 µm in diameter. Color in life is bright yellow or yellow to brown, red, violet or pink. The color of choanosome is always bright yellow. Specimens become dark brown upon contact with air. Preserved specimens have dark oxide coloration typical of the genus.

#### Skeleton

The skeletal structure is a regular tridimensional polygonal network of spongin fiber in a single category (Fig. 2 A). The fibers are amber in color, between 50 and 100 µm in thickness; they have typically a darker amber pith (from 25 to 58 µm in diameter), covering from 55 to 77% of the fiber diameter. Meshes are from 0.6 mm to 1 mm of opening. The structure of the fibers varies considerably from fibers with a thick core and a nearly imperceptible bark, to a regular core with a thick strongly striated bark (Fig. 2 B–E). Some fibers present short protuberances (Fig. 2 D).

#### Etymology

*Clathrum* (Greek) = lattice or grate, referring to the shape of an open latticework of anastomosed tubes.

#### Distribution and habitat

**Figure 3:**
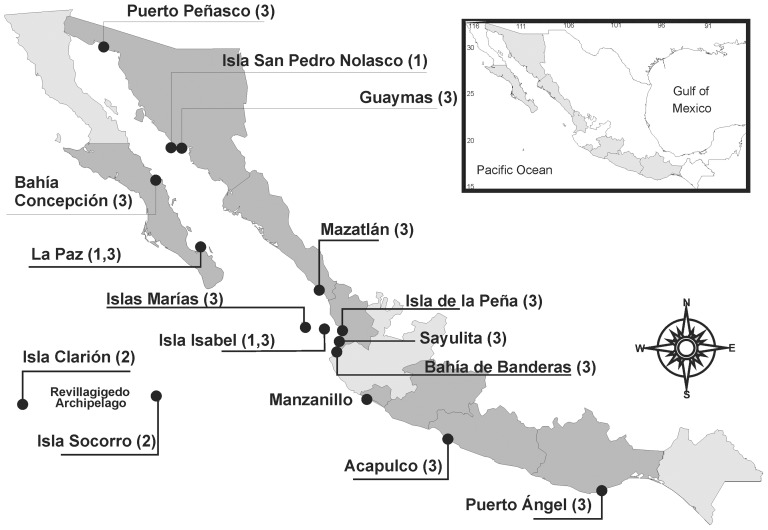
Sampling localities and distribution of *Aplysina* species along the Mexican Pacific Ocean. Numbers correspond to different species: (1) *Aplysina clathrata* sp. nov.; (2) *Aplysina revillagigedi* sp. nov.; (3) *Aplysina gerardogreeni*.

The species is distributed in tropical and subtropical coastal areas from the Mexican Pacific: Isla Espiritu Santo (La Paz, Baja California Sur), Isla San Pedro Nolasco (Sonora) and Isla Isabel (Nayarit) (Fig. 3). Specimens are common between 5 to 15 m deep, but the largest one was found at 20 m at the Isla Isabel. They are typical on rocks, in areas with high water flow and scarce sediment deposition.

#### Remarks

The combination of morphological characters and the presence of a regular tridimensional polygonal network of spongin fiber in a single category are typical of genus *Aplysina*. This is supported by molecular data of fragments from both genomes (see discussion). *A. clathrata* sp. nov. is clearly different from other *Aplysina* species by their typical latticework of anastomosed tubes, unique in the genus (see Table 1), which is very consistent and facilitates their identification. The only intraspecific variation seemingly ontogenetical is the more open clathrate structure in small specimens, and narrower to almost closed in the largest one (Fig. 1 A, B). The color varies from yellow in specimens from shallow water, to yellow and darker brown colorations in specimens living deeper.

**Table 1:** Comparative data of external morphology, skeletal characteristics and distribution of *Aplysina* species from Eastern Pacific and Atlantic Oceans and the Mediterranean Sea.

*Aplysina* species	External Characteristics (Color/form/oscula diameter)	Skeletal structure/Fibers/pith (diameter)	Distribution	Reference
**Eastern Pacific species**				
*A. clathrata* sp. nov. holotype MNCN 1.01/637	Yellow to yellow-brown/Sub-spherical clathrate-like/2–4 mm	Regular polygonal reticulation/50–100/25–58	Mexico	Present study
*A. revillagigedi* sp. nov. holotype MNCN 1.01/638	Green to green yellow/Cushon –shaped with oscula commonly organized on rims/1–5 mm	Irregular polygonal reticulation/70–130/50–110	Revillagigedo archipelago, Mexico	Present study
*A. gerardogreeni* Gómez & Bakus, 1992	Yellow and slightly pink, red or brown/Massive with oscular, lobular to tubular projections/3–5 mm	Regular polygonal reticulation/60–150/50–120	Mexico to Panama	Present study
*A. chiriquensis* Diaz et al., 2005	Pinkish-red or purple to bright yellow/Ramose departing from a stalk	Polygonal to oval reticulation/30–210/11–70	Panama to Galapagos Islands	[28]
*Verongia thiona* de Laubenfels, 1930	Lemon yellow with greenish tins/Encrusting from 4 cm thick/2–7 mm	Irregular, size average more than 1 mm/80–150/50–110	California EU.	[37]
**Western Atlantic species**				
*A. alcicornis* Pinheiro et al., 2007	Brown or yellow/Lamellar with oscula situated in depressions on the surface of the sponge/3 mm	Delicate and irregular network/50–142.5/15–82.5	Brazil	[30]
*A. caissara* Pinheiro & Hajdu, 2001	Bright yellow/Digitiform with oscula at the top of digits/1.5–4 mm	Delicate skeletal reticulation/26–77/17–60	Southeastern of Brazil	[30]
*A. cauliformis* (Carter, 1882)	Purple or light-yellow/Slender cylindrical branches with oscula on the projections	Irregular polygonal reticulation/22–190/7–115	Florida to Brazil	[30]
*A. cristagallus* Pinheiro et al., 2007	Bluish gray/Lamellar with oscula on the apex and sides	Delicate and irregular network/46–232/13–50	Brazil	[30]
*A. fistularis* (Pallas, 1766)	Yellow or brownish/Cylindrical, fusiform or slightly barrel-shaped tubes/7.5–8 mm	Irregular polygonal reticulation/37–275/10–60	Tropical Western Atlantic	[30]
*A. fulva* (Pallas, 1776)	Brownish to purplish yellow/Variable shaped but cylindrical, oscula over surface projections/0.5–2 mm	Irregular polygonal reticulation/21–275/10–72	Tropical Western Atlantic	[30]
*A. insularis* (Duchassaing & Michelotti, 1863)	Golden yellow/Short, irregularly outlined, stout, soft tubes with apical oscula/1 cm	Delicate and irregular network/35–125/12–37	Tropical Western Atlantic	[30]
*A. lactuca* Pinheiro et al., 2007	Yellowish-brown/Anastomosed lamellar form with oscula spread on all sides/1 mm	Delicate and irregular network/37–155/7–35	Northeastern Brazil	[30]
*A. lacunosa* (Lamark, 1814)	Bright yellow/Tubular with irregular grooves surface and a large apical pseudoscule/2.5 cm	Delicate and irregular network/7–196/7–37	Tropical Western Atlantic	[30]
*A. lingua* Pinheiro et al., 2007	Light yellow/Elongate lamellar with oscula over the entire surface/1 mm	Delicate and irregular network/37–192/10–35	Brazil	[30]
*A. muricyana* Pinheiro et al., 2007	Beige/Irregular polygonal tubes, laterally anastomosed with apical pseudoscula	Delicate and irregular network/38–126/8–50	Brazil	[30]
*A. orthoreticulata* Pinheiro et al., 2007	Beige/Digitiform with oscula on the sides of the sponge/1 mm	Orthogonal reticulation/100–307/10–40	Brazil	[30]
*A. pergamentacea* Hechtel, 1983	Laterally compressed lamellar, oscula located marginally in the sponge	Irregular polygonal reticulation/42–95/12–30	Brazil	[30]
*A. pseudolacunosa* Pinheiro et al., 2007	Brigth yellow and beige/From globular to tubular with apical pseudoscule/1.5 cm	Delicate irregular network/22–167/8–47	Brazil	[30]
*A. solangeae* Pinheiro et al., 2007	Yellow to yellow with purple stains/Lamellar in a semi-radial arrangement/1.5 mm	Delicate and irregular network/37–158/11–55	Northeastern Brazil	[30]
**Mediterranean species**				
*A. aerophoba* Nardo, 1843	Bright yellow/Digitate with apical oscula	Polygonal meshes/80–150/30–70	Mediterranean Sea	[29]
*A. cavernicola* (Vacelet, 1959)	Bright yellow/massive cushion-sapped with small irregular lobes topped by a osculum/1–3 mm	Regular polygonal network/18–65	Mediterranean Sea	[85]

***Aplysina revillagigedi*** **sp. nov.**

(Figs. 1 B, C; 2 F–H)

#### Material examined

Holotype: MNCN 1.01/638 Punta Tosca, Isla Socorro (Revillagigedo), 18°47′01″N, 111°02′42″W, 8 m depth, 05/08/2008. Paratypes: BMNH 2010.11.01.8 Punta Tosca, Isla Socorro (Revillagigedo), 18°47′01″N, 111°02′42″W, 8 m depth, 05/08/2008. LEB-ICML-UNAM-1236, Isla Clarion, Roca Norte (Revillagigedo), 18°47′14″N, 110°55′42″W, 4 m depth, 03/12/2005.

GenBank accessions: rDNA: JN596955; COI: JQ437580.

The ZooBank LSID: urn:lsid:zoobank.org:act:E7BEC0FA-038D-4C14-BAA6-88A19AD9498D.

#### Description

Cushion shaped to massive sponge (from 0.5 to 3 cm high), sometimes with rounded lobes from 0.8 to 1.2 cm high, covering area from 2 to 30 cm in diameter (Fig. 1 B, C). Surface is smooth; conules are evident only in preserved specimens, soft to the touch. Ostial pores from 16 to 67 µm in diameter. Oscules are very conspicuous, from 1 to 5 mm in diameter, circular to oval-shaped, regularly distributed on the surface, or linearly distributed on rims. They are slightly elevated from the surface. Consistence is flexible and firm. Ectosome is a thin membrane from 110 to 250 µm in thickness. Choanosome is dense with scarce canals from 86 to 170 µm in diameter, and little foreign debris. Color in life is commonly green, sometimes lemon yellow. Preserved specimens turn to the typical dark coloration of verongid sponges.

#### Skeleton

The skeletal structure is somewhat confuse but it is possible to distinguish an irregular polygonal tridimensional reticulation of sponging fibers in a single category (Fig. 2 F). Near the surface, fibers usually become ramified, and end in rounded tips (Fig. 2 G). Meshes are from 0.650 to 3.5 mm of opening. Fiber color varies in specimens from dark brown to amber; they are from 40 to 130 µm in thickness, with pith from 25 a 110 µm wide, which cover between 55 and 84% of the fiber diameter (Fig. 2 H).

#### Distribution and habitat

*Aplysina revillagigedi* sp. nov. is only known from Socorro and Clarion islands, and several rock pinnacles from Revillagigedo Archipelago (Mexican Pacific Ocean) (Fig. 3). The species is common in shallow clear waters, on vertical walls and rocky substrates exposed to high water movement. It was also found at 38 m.

#### Etymology

The specific epithet refers to the Revillagigedo archipelago where species is distributed.

#### Remarks

The only relatively similar species to *A. revillagigedi* sp. nov. in the Eastern Pacific area is *Aplysina gerardogreeni* which is characterized by having tubular lobules typically with an apical oscule, while in *A. revillagigedi* sp. nov. the tubes and lobes are uncommon, and usually have oscules organized on rims. In addition, the characteristic green color of *A. revillagigedi* sp. nov. has never been seen in *A. gerardogreeni*. Differences are also present at the skeletal level; *A. gerardogreeni* has a regular reticular spongin skeleton, while in *A. revillagigedi* sp. nov. skeletal structure is irregular with large meshes and fibers ending in a dendritic pattern. The species described as *Verongia thiona* de Laubenfels, 1930 from California (USA), is another close species to *A. revillagigedi* sp. nov. This species is an incrusting sponge with a few scattered oscules; skeletal structure is formed by large and irregular meshes some similar to *A. revillagigedi* sp. nov. However, *A. revillagigedi* sp. nov. is a cushion-shaped sponge with lobular formations, and oscula typically distributed on rims. In *V. thiona* the skeletal structure is composed by a reticulation of scattered fibers, whereas in *A. revillagigedi* sp. nov. the sponging structure is proportionally more abundant despite the presence of irregular meshes. In addition, the fibers in *A. revillagigedi* sp. nov. generally become dendritic near the surface, which was not described for *V. thiona*. Nevertheless, although there is not a formal decision, it has been suggested that *V. thiona* should be transferred to the genus *Aiolochroia* Wiedenmayer [35].

**Figure 4:**
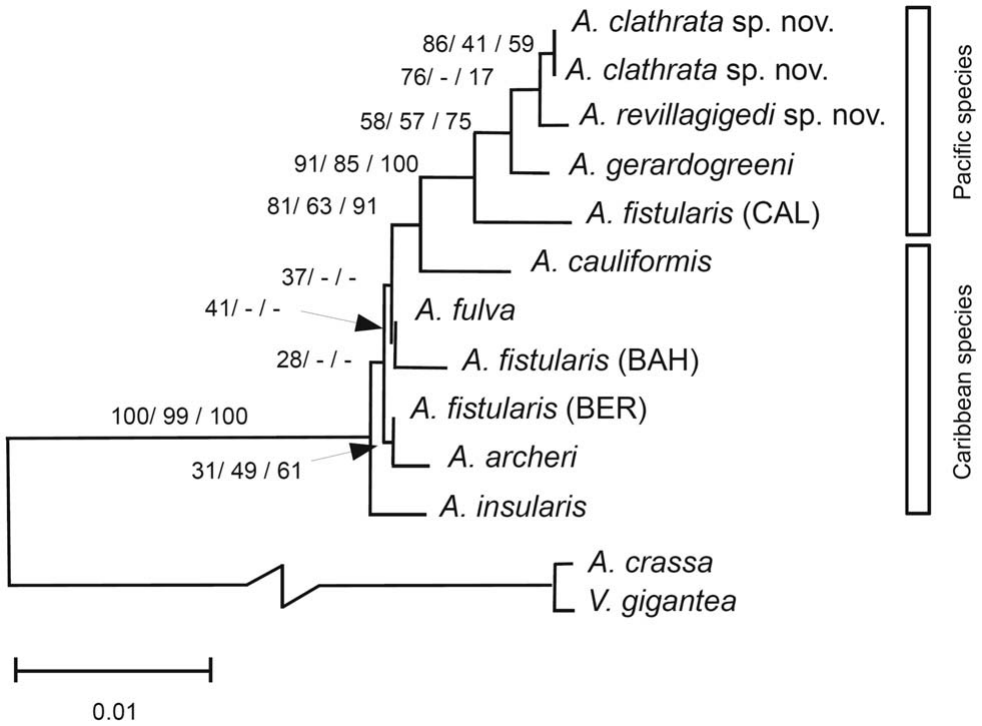
Neighbor-Joining phylogenetic reconstruction of Eastern Pacific and Caribbean species of the genus *Aplysina*. Numbers associated to each branch represent: NJ/MP bootstrap support values/Bayesian posterior probabilities (%). (-) indicates that a particular branch was not present in the MP or Bayesian reconstruction.

The presence of an irregular skeleton ending in ramified fibers at the surface resembles the genus *Suberea*, which is characterized by a coarse irregular dendritic fiber skeleton (see [29]). Although *Aplysina* and *Suberea* belong to different families (Aplysinidae and Aplysinellidae, respectively) both genera are quite similar. In fact it has been suggested that several *Aplysina* species require reexamination to confirm their generic assignment [29]. Despite the presence of a dendritic pattern of fibers near the surface in *A. revillagigedi* sp. nov., this species shares other typical characters with the genus *Aplysina*, such as a tridimensional network of large meshes formed by sponging fibers in a single category. The nuclear and mitochondrial genes confirm the position of the species in *Aplysina* (Fig. 4).

***Aplysina gerardogreeni*** Gómez & Bakus, 1992

(Figs. 1 E, F; 2 I, J)

#### Synonymy

*Aplysina gerardogreeni* Gómez & Bakus, 1992: 179, pl. 3, 4.

#### Material examined

LEB-ICML-UNAM-429, isla Pájaros 1 (Sinaloa), 23°15′29″N, 106°28′25″W, 10 m depth, 02/15/2002. LEB-ICML-UNAM-1002, cerro Pelón, isla Isabel (Nayarit), 21°51′21″N, 105°53′33″W, 21 m depth, 12/10/2003. LEB-ICML-UNAM-2027, Isla María Cleofas (Islas Marías), 21°17′59″N, 106°16′24″W, 3 m depth, 06/21/2008. LEB-ICML-UNAM-2028 Isla María Cleofas (Islas Marías), 21°17′59″N, 106°16′24″W, 3 m depth, 06/21/2008. LEB-ICML-UNAM-2042 Isla María Cleofas (Islas Marías), 21°17′59″N, 106°16′24″W, 8 m depth, 06/21/2008. LEB-ICML-UNAM-2073, Los Islotes, Isla Espíritu Santo (Baja California Sur), 24°35′57″N, 110°24′04″W, 6 m depth, 08/10/2009.

GenBank accessions rDNA: JN596958; mDNA-COI: JQ437578.

#### Description

Cushion shaped to massive lobulated sponge (1 to 5 cm high), characterized by having several tubular lobules (from 1 to 2.5 cm high and 1 cm wide), each one with an apical oscular aperture (Fig. 1 E, F). Specimens cover areas from 2 to 10 cm in diameter. The surface is smooth to minute conulose, with conula from 250–750 µm high. Surface is perforated by small ostial apertures from 40 to 150 µm in diameter. Oscules are circular or oval-shaped from less of 1 mm in smaller specimens to 3–5 mm in the largest one. The consistency is firm and slightly compressible. The ectosome membrane is an easily detachable dermis. The choanosome is cavernous, with channels 40 to 160 µm in diameter. Color in life is very variable, from bright to dull yellow and some parts are slightly pink, red or brown; it turns dark brown or purple in contact with the air.

#### Skeleton

Skeletal structure consists of a single class of fibers, which form a regular tridimensional polygonal network with meshes from 1.1 to 1.9 mm wide (Fig. 2 I). The fibers are smooth; color varies in specimens from amber or dark. They are from 60 to 150 µm in diameter, with darker pith from 50 to 120 µm diameter, covering between 76 and 95% of the fiber (Fig. 2 J). Near the surface the fibers usually bifurcate and form the conular surface ending in rounded tips.

#### Distribution and habitat

The species was described in the Mexican Pacific Ocean [45], and later reported in Panama [46]. *A. gerardogreeni* is the most common verongid species along the Mexican Pacific coast. Specimens were found in Baja California Sur, Sonora, Sinaloa, Nayarit, Jalisco, Michoacán, Guerrero and Oaxaca (Fig. 3, see [45]). The species is found attached to hard substrates such as rocks, coral rubble or artificial substrates from the intertidal to 30 m deep.

### Identification key of the Eastern Tropical Pacific Aplysina -species

Pedunculate sponge……………………………… *A. chiriquensis*

Clathrare like sponge………………………..*A. clathrata* sp. nov.

Cushion- shaped to massive species…………………………..2

2 *Aplysina* commonly yellow, with lobules and/or tubes topped by an oscule ………………………………….…….*A. gerardogreeni*

2 *Aplysina* usually green, without tubes, and with oscula lineally on rims; ……………………….……………*A. revillagigedi* sp. nov.

### Chitin analyses

**Figure 5:**
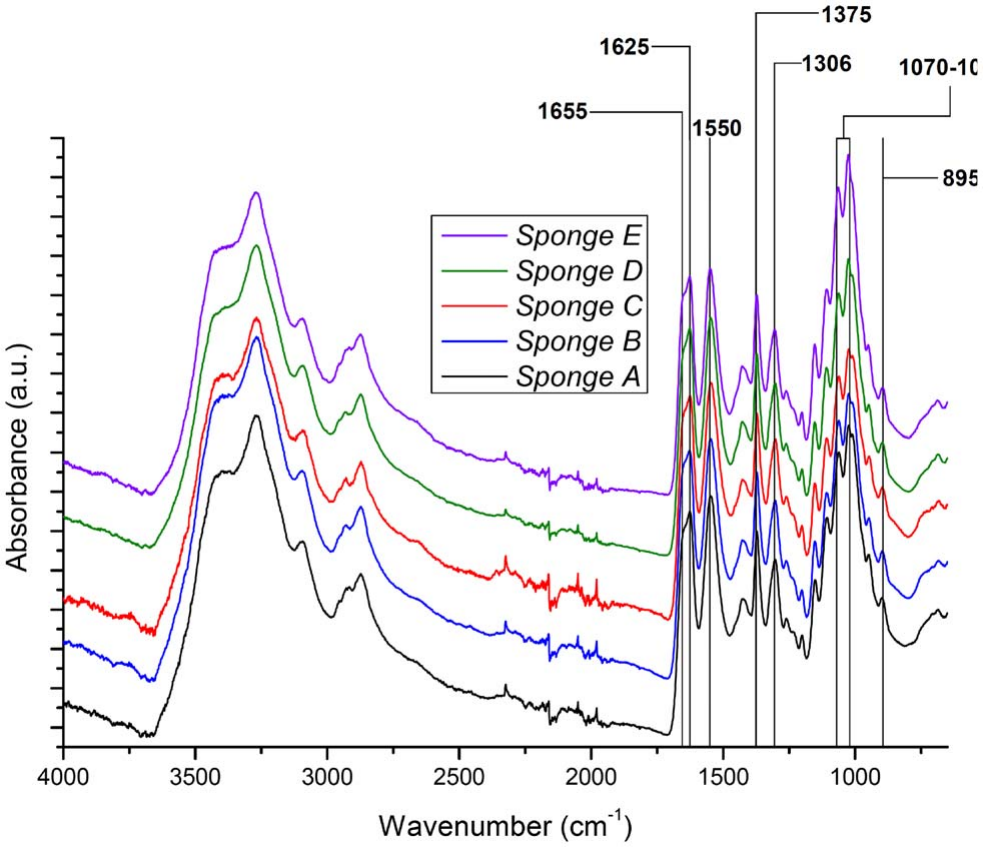
Results of the infrared spectroscopy of purified skeletons. *Aplysina fulva*; A) *A. gerardogreeni*; B) *A. clathrata* sp. nov.; C) *A. revillagigedi* sp. nov.; D) *Suberea azteca. Apysina fulva*, and *Suberea azteca* (unpublished data) are only included for comparative purposes.

**Figure 6:**
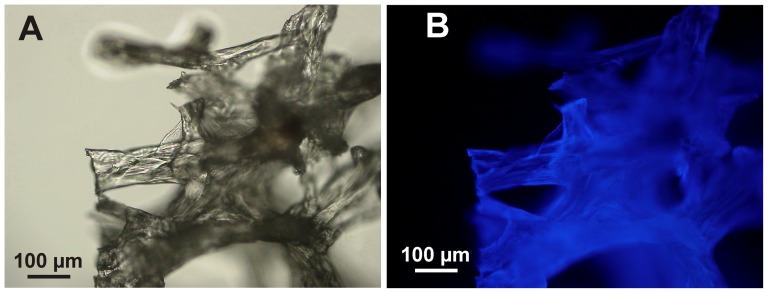
Demineralized and purified skeletal fibres of *Aplysina revillagigedi* sp. nov. A) Light microscopy image; B) Show intensive fluorescence after Calcofluor White staining for chitin. The light exposure time for fluorescence microscopy was 1/1000 s.

Chitin as main structural component of the *Apysina* spp. has been unambiguously identified using FTIR (Fig. 5) and specific Calcofluor White staining [23] (Fig. 6). The Morgan-Elson assay for the determination of N-acetyl-D- glucosamine (NAG) in chitin-based scaffolds indicated some variability among the species studied. The mean amounts of NAG have been estimated as 350 (±10 S.D.) µg/mg, 285 (±10 S.D.) µg/mg and 375 (±10 S.D.) µg/mg of dry skeleton for *A. revillagigedi* sp. nov., *A. gerardogreeni*, *A. clatrata* sp. nov., respectively.

### Molecular analyses

#### Genetic variation and inter-specific divergence

The origin of our sequences as Porifera was confirmed by BLAST searches, thereby discarding the possibility of contamination. Comparisons with other *Aplysina* sequences from GenBank revealed high similarities (>90%) for both genes (COI mtDNA and ITS1-5.8-ITS2 rDNA). New sequences were deposited in GenBank (Table 2).

**Table 2:** Accession numbers of the specimens sequences, vouchers and DNA sequences analyzed.

Species	Collection/museum accession number	Locality	GenBank accession (rDNA/COI)
**Eastern Pacific**			
*Aplysina clathrata* sp. nov.	MNCN 1.01/637	Isla Isabel, México	JN596956/JQ437579
	BMNH 2010.11.01.7	Isla Isabel, México	JN596957/JQ437579
	LEB-ICML-UNAM-2022	Isla Isabel, México	JN596956/JQ437579
	LEB-ICML-UNAM-2023	Isla Isabel, México	JN596956/JQ437579
	LEB-ICML-UNAM-2072	Isla Espíritu Santo, México	JN596956/JQ437579
*Aplysina revillagigedi* sp. nov.	MNCN 1.01/638	Isla Socorro, México	JN596955/JQ437580
.	BMNH 2010.11.01.8	Isla Socorro, México	JN596955/JQ437580
	LEB-ICML-UNAM-2024	Isla Socorro, México	JN596955/JQ437580
	LEB-ICML-UNAM-2025	Isla Socorro, México	JN596955/JQ437580
*Aplysina gerardogreeni*	LEB-ICML-UNAM-429	Isla Pájaros México	JN596958/JQ437578
	LEB-ICML-UNAM-1002	Isla Isabel, México	JN596958/JQ437578
	LEB-ICML-UNAM-2027	Isla María Cleofas, México	JN596958/JQ437578
	LEB-ICML-UNAM-2028	Isla María Cleofas, México	JN596958/JQ437578
	LEB-ICML-UNAM-2042	Isla María Cleofas, México	JN596958/JQ437578
	LEB-ICML-UNAM-2073	Isla Espíritu Santo, México	JN596958/JQ437578
*Aplysina fistularis*		California EU	AY591792.1
**Caribbean**			
*Aplysina insularis*		Bahamas	AY591794.1
*Aplysina fulva*		Bahamas	AY591793.1
*Aplysina fistularis*		Bermuda	AJ621545.1
*Aplysina fistularis*		Bahamas	AY591791.1
*Aplysina archeri*		Bahamas	AY591788.1
*Aiolochroia crassa*		Bahamas	AY591798.1
*Verongula gigantea*		Bahamas	AY591797.1

**Figure 7:**
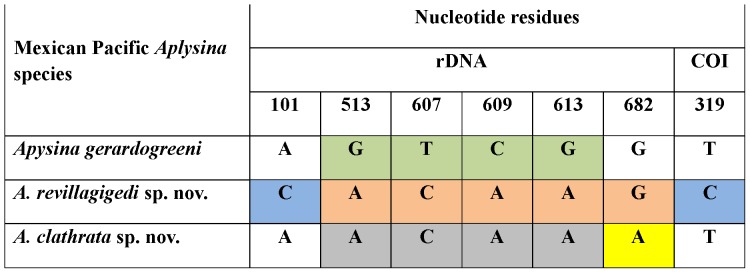
Diagnostic nucleotides and groups of nucleotides following Davis and Nixon [**48**] for three species of *Aplysina* from the Mexican Pacific. Single-nucleotide pure diagnostic characters are individually color-coded for each species (green: *A. gerardogreeni*, blue: *A. revillagigedi*, and yellow: *A. clathrata*); additional composite diagnostic combinations are indicated for *A. revillagigedi* (orange) and *A. clathrata* (gray). ITS1-5.8S-ITS2, nuclear ribosomal DNA; COI, mitochondrial cytochrome oxidase subunit I. Nucleotide residues refer to the individual alignments of ITS1-5.8S-ITS2 and COI sequences (GenBank accessions: ITS1-5.8S-ITS2 rDNA JN596955–58 and COI mtDNA JQ437578–80). Nucleotide 101 is ITS1 and the rest are ITS2.

We aligned 523 bp of the mtDNA COI gene of *A. gerardogreeni*, *A. clathrata* sp. nov. and *A. revillagigedi* sp. nov. (Table 2). COI sequences were extremely conserved showing only one segregating site among species (Fig. 7). The variable site was diagnostic for *A. revillagigedi* sp. nov. Hence, no subsequent analyses were carried out on these data.

After clipping low quality end-reads, the multiple alignments spanning the ITS1-5.8S-ITS2 genes of the rDNA encompassed 705 bp from 11 specimens of the three species of *Aplysina* in the Mexican Pacific (Table 2).

Based on the identical result obtained with different amplification strategies from the same specimens, i.e. entire fragment and ITS1 and 2 separately, we inferred the absence of intragenomic rDNA polymorphisms in the analyzed organisms.

We detected intraspecific ITS-1 rDNA polymorphisms only in *A. clathrata* sp. nov., in which two haplotypes were found (*h* = 0.67 and π = 0 -nucleotide differences correspond to indels-). *A. revillagigedi* sp. nov. and *A. gerardogreeni* were monomorphic.

Given the absence of intraspecific nucleotide substitutions, we treat interspecific polymorphic sites as provisionally diagnostic. Following Davis and Nixon (1992) [48] character-based diagnosis, we found four diagnostic nucleotides for *A. gerardogreeni* (ITS1-2), whereas *A. clathrata* could be diagnosed by two nucleotides (ITS1-2, COI) and an additional diagnostic combination of five nucleotides (ITS1-2). *A. clathrata* could be diagnosed by a single nucleotide and a diagnostic combination of four nucleotides (ITS1-2) (Fig. 7).

Interspecific polymorphisms of the nuclear ITS regions, including additional *Aplysina* species from GenBank, revealed a total of 35 variable sites, 12 of which were parsimony- informative. Pair-wise sequence divergence (uncorrected p-distance) between *A. clathrata* sp. nov. and *A. revillagigedi* sp. nov., was 0.288%. In contrast, the divergence between *A. clathrata* sp. nov. and *A. gerardogreeni* and between *A. revillagigedi* sp. nov. and *A. gerardogreeni* was 0.719% (Table 3). The Eastern Pacific individual identified as *A. fistularis* by Schmitt et al. (2005) is more similar to *A. clathrata* sp. nov. (p-distance = 1.007%) than to the rest of the species (p-distance>1.29%).

**Table 3:** Pairwise ITS1-5.8S-ITS2 rDNA genetic divergence (% uncorrected p-distance) between *Aplysina* species, from Eastern Pacific and Caribbean.

	*A. gerardogreeni*	*A. revillagigedi* sp. nov.	*A. clathrata* sp. nov.	*A. fulva*	*A. fistularis* (CAL)	*A. fistularis* (BER)	*A. fistularis* (BAH)	*A. archeri*	*A. cauliformis*
***A. revillagigedi*** ** sp. nov.**	0.719								
***A. clathrata*** ** sp. nov.**	0.719	0.288							
***A. fulva***	1.295	1.439	1.439						
***A. fistularis*** ** (CAL)**	1.727	1.295	1.007	1.583					
***A. fistularis*** ** (BER)**	1.439	1.583	1.583	0.144	1.727				
***A. fistularis*** ** (BAH)**	1.727	1.871	1.871	0.432	2.014	0.576			
***A. archeri***	1.727	1.871	1.871	0.432	2.014	0.288	0.863		
***A. cauliformis***	1.727	1.871	1.871	1.007	2.014	1.151	1.439	1.439	
***A. insularis***	1.871	2.014	2.014	0.576	2.158	0.719	1.007	1.007	1.583

#### Phylogenetic analyses

All methods of phylogenetic reconstruction strongly supported the monophyly of Eastern Pacific and Caribbean *Aplysina* species relative to *Aiolochroia crassa* and *Verongula gigantea* (Fig. 4). The rDNA phylogeny of *Aplysina* revealed a phylogeographic pattern. A well-supported (91% NJ, 85% MP bootstrap, 100% posterior probability) monophyletic clade grouped all Eastern Pacific sequences from this and previous studies, including a sequence from a putative *A. fistularis* specimen collected in California (Schmitt et al. 2005) [42] (see discussion). *A. cauliformis* from the Caribbean was consistently placed as sister taxon to the Pacific species. In all trees, *A. gerardogreeni* appears as sister taxon to *A. revillagigedi* sp. nov. and *A. clathrata* sp. nov., strong support for this relationship is mostly present in the NJ tree (Fig. 4). A second set of Caribbean sequences from previous studies (excluding *A. cauliformis*) was paraphyletic and very poorly resolved. In the single most parsimonious tree (length = 165, not shown) *A. fulva* and the two Caribbean *A. fistularis* sequences grouped as a sister clade to [*A. cauliformis*, Pacific species]; whereas *A. archeri* and *A. insularis* formed a basal sister clade to the rest of *Aplysina*. However, these relationships were not supported by high bootstrap levels.

## Discussion

### Taxonomy of Eastern Pacific Aplysina-species

Although 84 species of *Aplysina* have been described so far, only 44 are considered valid (Porifera Data Base [47]), and from these, only 23 remain clearly valid today [28], [30]. The rest are considered unrecognized, due to the lack of good descriptions and the poor preservation of type material [28].

*Aplysina* has a circumtropical distribution, but the status of the species recorded in the Indo-Pacific remains unclear and needs to be revised [28]. The Western Atlantic is the region with the highest diversity (16 species), whereas the Mediterranean Sea has only two known species. In the Eastern Pacific only two species; *Aplysina gerardogreeni* and *A. chiriquensis* should be hitherto considered valid. Others, such as *A. aurea* (by [48], [49]), *A. lendenfeldi* and *A. fulva* (by [50]), and *A. fistularis* (by [51]), must be considered invalid due to absence of detailed morphological description and no specialized identification. The latter should not be confused with *A. fistularis sensu* Green 1977 [52], which is a valid record from Veracruz, Gulf of Mexico (Atlantic), but not from the Pacific as was considered by Diaz et al. (2005) [28].

Other Eastern Pacific species related to *Aplysina* are *Suberea azteca* (Gomez & Bakus 1992) described in Mexico, and *Verongia thiona* de Laubenfels, 1932 described in California (USA). *Suberea azteca* was originally described as *Aplysina*, but was later transferred to the genus *Suberea* based on its skeletal fiber structure [53]. *V. thiona* should be attributed to the genus *Aplysina* due to the synonymy of *Verongia* with *Aplysina* [35]. However, the lack of a detailed original description hinders the clarification of its taxonomic status, which has been suggested to correspond to the genus *Aiolochroia* Wiedenmayer, 1977 (see [47]), but no formal amendment has been published. According to de Laubenfels (1932) specimens of *V. thiona* are abundant in Laguna Beach California USA (type locality), and moderately common in the intertidal areas of southern California. A specimen of *Aplysina* (identified as *A. fistularis*) was collected in La Jolla, Southern of California, near *V. thiona*'s type locality (less than 100 km away), and was sequenced by Schmitt et al. (2005) [42]. Our phylogenetic analyses place this sequence within the Eastern Pacific clade of *Aplysina* (Fig. 4). Therefore, we hypothesize that the specimen sequenced by Schmitt et al. (2005) may correspond to *V. thiona* and not to *A. fistularis*, (possibly endemic to the Caribbean). If the identity of this specimen can be verified as such, then our molecular phylogenetic analyses support the synonymy of *Verongia* and *Aplysina*.

The Eastern Pacific *Aplysina* species are easily distinguishable from each other. *A. chiriquensis* is characterized by having a pedunculate shape. *Aplysina clathrata* sp. nov. is also well differentiated by its typical clathrate-like morphology, which has never been described for a species in the genus. *Aplysina gerardogreeni* may be more similar to *Aplysina revillagigedi* sp. nov. because both may have cushion-shaped form. However, *A. gerardogreeni* is characterized by a completely lobular surface with an apical oscule on lobes and tubes, whereas lobules are uncommon in *A. revillagigedi* sp. nov. and oscula are usually organized on rims.

### Molecular Systematics of *Aplysina*

The order Verongida is a cohesive group of marine sponges whose systematics has been traditionally based on morphology, and more recently on biochemical and genetic data [43]. Even though morphological characters have not been useful to resolve its phylogeny [43], molecular data have supported the monophyly of the group in several gene trees (see [1], [2], [43], [54]), and have shown to be useful to address relationships down to the species level [43].

Our phylogenetic analyses have also supported the monophyly of *Aplysina* (Fig. 4), as shown in analyses of 18S and ITS-2 in species of Verongida [42], [43]. ITS spacers have also been useful for species-level discrimination in other sponges (e. g. in genera *Dysidea*, *Axinella*, among others) [55], [56]; and in the genus *Aplysina* this gene region produced a robust phylogeny at the species level [43]. Being a multicopy gene cluster, rDNA has shown elsewhere evidence of intragenomic variation in *Aplysina* [41], [57]. However, our genetic analyses of Mexican sponges did not reveal this kind of variation. Nevertheless, the presence of intragenomic polymorphisms does not preclude the usefulness of the analyzed rDNA fragment as a suitable genetic marker; but it calls for caution in the interpretation of the patterns of diversification [10], [57], [58].

The phylogenetic relationships of Caribbean and Pacific *Aplysina* species had already been mentioned by Schmitt et al. (2005) [42] who have also included sequences from the Mediterranean in their analyses. They suggest a biogeographical hypothesis where *Aplysina* could have a Tethyan origin resulting in a Mediterranean and a Caribbean-Pacific clade. The subsequent and recent adaptive radiation of all extant Caribbean and Eastern Pacific *Aplysina*, explain their high degree of genetic similarity [42]. Our results clearly show a monophyletic Pacific clade vis-à-vis a paraphyletic group of poorly resolved Caribbean sequences, suggesting a single Pacific ancestor and that speciation may have ensued following the rising of the Isthmus of Panama, when amphi-american faunas were separated. Our gene tree also reveals that *A. cauliformis* (a Caribbean species) represents a lineage more closely related with the Eastern Pacific clade, which may be interpreted either as an extant representative of the lineage that gave rise to the Pacific clade or a possible invasion of the Caribbean by an early Pacific branch.

Eastern Pacific species were grouped in general accordance with the taxonomic hypothesis based on morphological characters. *A. revillagigedi* is an insular species separated from the rest and is also a divergent lineage among the Eastern Pacific *Aplysina*. On the other hand, *A. clathrara* and *A. gerardogreeni* are sometimes sympatric and their genetic divergence is less pronounced.

### DNA Barcoding

Although the mtDNA COI gene has been a valuable marker to differentiate many metazoan species and is the marker of choice in the Barcoding of Life initiative [4], in general it has shown very low levels of variation in diploblastic organisms such as sponges and corals [8], [9], [10]. The virtual lack of variation in our COI sequence supports the shared ancestry of the mitochondrial lineages. On the other hand, it is not entirely devoid of power for species discrimination, given that the only polymorphic nucleotide resulted in a mutation that appears to be diagnostic for *A. revillagigedi* sp. nov. vis-à-vis the other two sequenced species (*A. clathrata* sp. nov. and *A. gerardogreeni*) (Fig. 7). Previous analyses of COI sequences failed to resolve seven species of *Aplysina* from the Mediterranean and Caribbean. The Caribbean *A. insularis*, *A. cauliformis*, *A. fulva* and *A. fistularis* possessed identical COI sequences whereas the Mediterranean *A. aerophoba* and *A. cavernicola* differed by one base pair [41], [42]. The presence of a single polymorphic site in the COI gene was also found to be congruent with morphological differences observed among species, as it is the case in this study [41], [59]. However, our results and others confirm that in some instances COI-DNA barcoding is an efficient method for the discrimination of sponge taxa at least to the generic level [60], [61].

In addition, COI-DNA barcoding for sponges may be complemented by other molecular markers for a finer resolution. Non-coding intergenic transcribed spacers ITS-1 and ITS-2 from rDNA have been successfully used in micro- and macro-evolutionary studies due to their faster rate of evolution relative to functional genes [57], [62]. They have been used in population genetic studies, but among sponge taxa mainly to infer phylogenies [42], [43], [56], [63]–[66]. Intra-genomic variation in the ITS region has been found in *Aplysina* from the Caribbean and Mediterranean, suggesting that this region may not be entirely appropriate for fine-scale population studies [41]. However, we found no evidence of intra-individual polymorphisms in the specimens analyzed in this study. In our analyses, the ITS region provided enough variation for Eastern Pacific species differentiation (Figs. 4, 7) and, even though it has shown intra genomic polymorphisms elsewhere, it remains a good candidate for DNA barcodes of plants and animals [67].

### The skeletons: chemical chitin composition

The absence of a mineral skeleton in *Aplysina* spp. has represented a challenge for species identification, and for the estimation of the phylogenetic relationships in the group. The chemical analysis showed that alpha-chitin is present in all the species studied, which is highly relevant and strengthens the hypothesis that all species of *Aplysina* share with other members of the order Verongida the presence of chitin-based scaffolds [24] as a synapomorphy supporting the monophyly of Verongida.

The chemical analyses of the fibers indicated some variability of N-acetyl-D- glucosamine concentration among the Eastern Pacific species of *Aplysina*, which were also similar to the one found in Atlantic and Mediterranean *Aplysina*-species [24]. Spectra were also very similar between the Mexican *Aplysina*, and the one obtained previously from the skeleton of *A. fulva* [24], but each species revealed differences in absorbance at different wave numbers (Fig. 5), which could have taxonomical importance. Nevertheless, the significance of these differences for species distinction is still unclear and more research may be needed to evaluate their possible use in *Aplysina* taxonomy.

### Integrative taxonomy in *Aplysina*

The systematics of Porifera is traditionally based on skeletal characteristics (spicules and fibers, and their arrangement on sponge body), but the paucity, simplicity and plasticity of these characters are the principal limitation in species determination and phylogenetic interpretation [68]. Alternatives to traditional taxonomy have been created as a result of technological advances (e. g. chemotaxonomy and molecular taxonomy). However, in several cases they act independently and sometimes differing to traditional conceptions. Currently the major approximation to species identification could be the combined use of morphological and molecular data, both characteristics could give an efficient way to evaluate the taxonomic status of species [17], [18].

The importance of the integrative approach used in this study is emphasized by the difficulty in the interpretation of phenotypic variation sometimes encountered in sponge systematics. Although the analyzed species can be readily recognized as *Aplysina*, they also show some characteristics that could confuse the taxonomic determination. For instance, *A. clathrata* sp. nov. has an external morphology previously undescribed in *Aplysina*. The external surface is perhaps more similar to species of the genus *Aiolochroia*, which is characterized by rounded tubercles surrounding depressions giving it an overall polygonal appearance. In addition, *A. clathrata* sp. nov. also possesses fibers with nodules and short protuberances characteristic of *Aiolochroia* (see [29]). However, our genetic results are emphatic that these species are not close to *Aiolochroia*, which was used as an outgroup to root our phylogenetic reconstruction (Fig. 4). *A. revillagigedi* sp. nov. has an irregular skeletal structure which becomes dendritic towards the surface, resembling the genus *Suberea* (Verongida: Aplysinellidae) which is characterized by a coarse irregular dendritic fiber skeleton (see [29]). However, in both cases, the molecular analyses support that they are well assigned to the genus *Aplysina* and therefore shed light on the plasticity of the morphological characters in the group.

The use of an “Integrative taxonomy” for species delimitation was pointed out separately by several authors [11], [68], [69]. The proposal was quickly adopted and several approaches have been presented for how the integration of different kinds of characters should be addressed (e.g., [3], [11], [13], [17], [18]). Among these, authors have argued about the importance of congruence between morphological and molecular characters (i.e., “integrative taxonomy by congruence”, [3]), which is well exemplified by the taxonomic circle [12]. This procedure illustrated that congruence of two or more taxonomic characters is an important factor in species identification, assuming that increased support for distinction arising from independent data sets decreases the likelihood of erroneous delineations of novel taxa. Operationally, the circle represents the experimental routes (e.g., DNA, morphology, reproduction, ecology and geography) involved in taxonomic inference to objectively corroborate taxonomic hypotheses. The only way to delineate a new taxon is to break out of the circle following an initial hypothesis after visiting independent lines of evidence placed on the perimeter of the circle [12].

**Figure 8:**
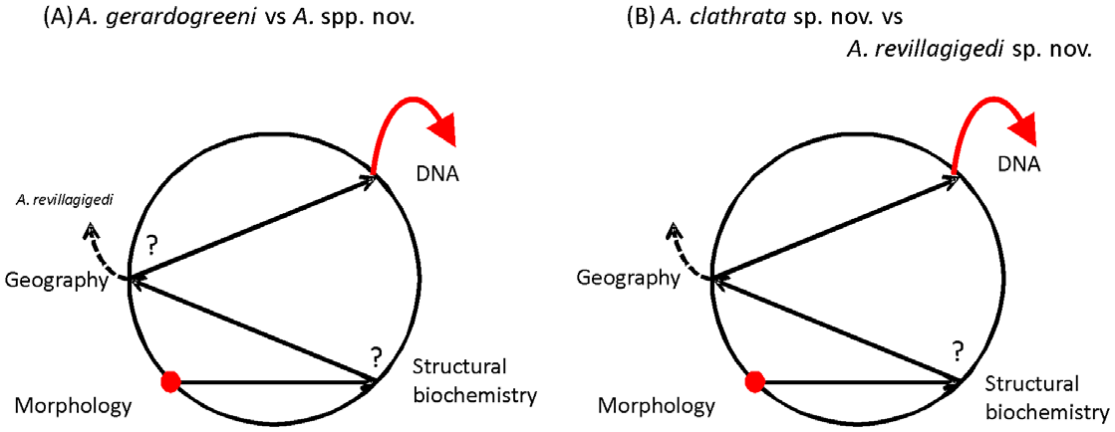
Delineation of new *Aplysina* species from the Mexican Pacific under the framework of the taxonomic circle [**12**]. A) The first hypothesis consists in differentiating the new species *A. clathrata* sp. nov. and *A. revillagigedi* sp. nov. from *A. gerardogreeni*, based on an initial morphological distinction. For this, ecological and structural biochemical evidence per se are not conclusive to break-out of the circle. Geography could allow breaking-out the circle for *A. revillagigedi* sp. nov. (dashed arrow), an insular endemic, but not for both. However the integration of the molecular data in the form of diagnostic characters as well as quantitative divergence and phylogenetic analyses provide congruent information with the rest allowing to breakout of the circle; B) The second hypothesis consists in differentiating the two new species. For this geographical distribution (continental vs. insular) could help to break out of the circle (dashed arrows) but structural biochemistry remains inconclusive. However, the addition of molecular evidence indicating the genetic distinction and diagnostic characters in the nuclear and mitochondrial genomes provide the unequivocal evidence of reproductive isolation, allowing breaking out of the circle.

Following this approach, the delineation and distinction of both *A. clathrata* sp. nov. and *A. revillagigedi* sp. nov. from *A. gerardogreeni* comes from morphology, which provides the initial hypothesis and the most conspicuous evidence to discriminate the three *Aplysina* species (Fig. 8). However, the difficulty in morphological taxonomy of *Aplysina* is mainly due to the lack of diagnostic characters and high phenotypic plasticity of the individuals, making it hard to establish the boundaries between species. So it is very important to incorporate other characters, and corroborate whether they are congruent with morphology in species discrimination. In addressing the distinction of the new species from the widespread and valid *A. gerardogreeni*, geographical information could help “break out of the taxonomic circle” for *A. revillagigedi* sp nov., by geographical isolation (Fig. 8 A). This species of *Aplysina* is endemic to the Revillagigedo Archipelago, where the other two species are absent. Unlike chitin spectroscopic profiles, which remain inconclusive at the moment and more research is needed to evaluate their possible use in *Aplysina* taxonomy, nuclear ITS1-2 rDNA sequences provide diagnostic characters as well as quantitative divergence and phylogenetic analyses congruent with the other evidence allowing to break out of the circle for the distinction of the new species from *A. gerardogreeni* (Fig. 8 A). In a second hypothesis, the distinction between the two new species, *A. clathrata* sp. nov. and *A. revillagigedi* sp. nov. is also prompted by morphological differentiae (Fig. 8 B). Following the circle, the distinction is clearly supported by geographical distribution (continental vs. insular), which could help to breakout of the circle. However, genetic distinction at the DNA level and diagnostic nucleotides in nuclear (ITS1-2) and mitochondrial (COI) genomes provide unequivocal evidence of reproductive isolation, also allowing to break out of the circle (Fig. 8 B).

Hence, our research supports that the best approach for species identification of Porifera is through of integrative use of taxonomic characters that can reciprocally shed light in the evolution of each other. In the case of Eastern Pacific *Aplysina*, integrated information of molecular and morphological characters, in addition to geographic and, to a lesser extent, chemical information provided congruent support to the taxonomic distinctions.

To date, there are several studies emphasizing the importance of combined traditional morphological taxonomy with molecular markers in species identification [40], [70]–[72]. They have been useful in clarifying the systematics of several sponge groups [59], [73]–[75]. In fact the recent initiative Sponges Barcoding Project http://www.spongebarcoding.org (see [40]) not only makes available DNA-barcoding of sponges but it also includes detailed morphological characteristics of analyzed species. However in spite of these important approaches favoring an integrative taxonomy in Porifera, a protocol facilitating the unification of criteria is missing. The proposed taxonomic circle is a good example that could be adopted in the taxonomy of Porifera

Our results constitute one of the first approximations to integrative taxonomy, phylogeny an evolutionary biogeography of Eastern Pacific marine sponges (see also [63]). Subsequent information about distribution patters of sponges will help clarify the evolutionary process and speciation mechanisms of the Eastern Pacific sponge faunas.

## Materials and Methods

### Specimens

Ninety-six specimens were collected by scuba diving and snorkeling in 43 localities along the Mexican Pacific Ocean (Fig. 3). Some were used for both morphological and molecular analyses (Table 2). For morphological analyses specimens were fixed in 4% formalin for 24 h and later transferred to 70% ethanol for storage whereas tissue samples for molecular analyses were preserved in 96% ethanol and stored at −10°C. Biological material was deposited in the *Colección de Esponjas del Pacífico Mexicano* (LEB-ICML-UNAM), of the Instituto de Ciencias del Mar y Limnología, UNAM, in Mazatlán (México). Type material has been deposited in the Museo Nacional de Ciencias Naturales in Madrid (Spain) (MNCN), and in the British Museum of Natural History (BMNH) (London).

### Morphological analyses

External morphology and fiber characteristics were recorded for each species. Fiber preparation for light microscopy involved the chemical digestion of surrounding tissue by incubation in a clearing solution (1/3 of distilled water, 1/3 hydrogen peroxide H_2_O_2_, and 1/3 of ammonia). Fragments were agitated daily, and the solution replaced every 24 hours until the fibers were cleared of tissue [76]. Fiber measurements were obtained from a minimum of 25 portions chosen randomly for each specimen. They are given in fiber and pith width, and percentage of pith with respect to the entire fiber. Fibers, pith and skeletal measurements are given in micrometers or millimeters and mean values are presented in brackets. Anatomical terms follow Boury-Esnault & Rützler [77].

### Chemical analyses

Chitin has been extracted from ca. 2×3 cm fragments of the sponges (*A. revillagigedi* sp. nov. n = 3, *A. gerardogreeni* n = 3, *A. clatrata* sp. nov. n = 3), by subjecting them to chemical treatment fully described previously [23], [24]. To remove other compounds from the chitin, the sample underwent a series of extraction steps to remove impurities. These extractions included step-by-step treatment as follows: an acidic extraction, an alkali-based extraction, an optional hydrogen peroxide treatment, and washing steps using distilled water before and after each treatment step.

In order to estimate N-acetyl-d-glucosamine contents (NAG), 6 mg of each dried skeleton were pulverized to a fine powder in an agate mortar [24]. Preparation of colloidal chitin from a crab alpha-chitin standard (Sigma) was performed according to Boden et al. (1985) [78]. The Morgan-Elson assay was used to quantify the N-acetyl-d-glucosamine released after chitinase treatment as described previously [78].

To elucidate the particular location of chitin in investigated samples, we used Calcofluor White (Fluorescent Brightener M2R, Sigma). Samples were placed in 0.1 M Tris-HCl at pH 8.5 for 30 min, then stained using 0.1% Calcofluor White solution for 30 min in darkness, rinsed five times with deionized water, dried at room temperature, and finally observed using fluorescence microscopy.

Fourier Transform Infrared spectroscopy was carried out using FTIR Bruker IFS 66/s with the following parameters: spectral resolution: 2 cm^−1^; scans: 500 scans; spectrum: 3900-1000 cm^−1^; aperture: 1 mm; MCT Detector; mirror rate: 40 KHz.

Light and fluorescence microscopy observations were carried out using Digital fluorescence microscope BZ-8000 (Keyence).

### Molecular analyses

#### DNA purification, amplification and sequencing

Total genomic DNA was extracted using standard proteinase K digestion in CTAB extraction buffer and purified with a LiCl salting- out protocol, followed by organic extraction using chloroform–isoamyl alcohol, and subsequent ethanol precipitation [79]. Subsequently, the mtDNA COI gene and the nuclear rDNA encompassing the ITS1-5.8S-ITS2 gene region were PCR-amplified and sequenced from 11 specimens of the different species (Table 2).

The mtDNA gene was initially amplified using invertebrate universal primers [80] yielding inconsistent results. Therefore, we designed nested primers (COXI_AplysinaF 5′-TTG CTG GTA TGA TAG GAA CAG-3′ and COXI_AplysinaR 5′-TGA TAT AAA ATT GGG TCC-3′), which were used to amplify around 523 base pairs (bp) of the COI gene. PCR reactions (25 µl) consisted of 8.30 µl dH_2_O (sterile MilliQ), 9 µl dNTPs (0.5 mM each), 0.50 µl each primer (10 µM), 2.50 µl 10× PCR buffer (20 mM MgCl_2_), 1 U *Taq* DNA polymerase, 1 µl BSA and 3 µl of genomic DNA (ca. 50–100 ng). Thermal cycling conditions were: initial denaturalization of 94°C for 2 min, and 35 cycles of 94°C for 30 sec, 50°C for 30 sec, 72°C for 1 min, and a final extension of 72°C for 5 min.

The rDNA gene region was amplified using universal primers (ITS4 5′-TCC TCC GCT TAT TGA TAT GC-3′ and ITS5 5′-GGA AGT AAA AGT CGT AAC AAG G-3′) [81]. PCR reactions (25 µl) consisted of 9.30 µl of dH_2_O (sterile MilliQ), 9 µl of dNTPs (0.5 mM each), 1 µl of each primer (10 µM), 2.50 µl of 10× PCR buffer (20 mM MgCl_2_), 1 U *Taq* DNA polymerase, and 2 µl of genomic DNA (ca. 40–67 ng). Thermal cycling conditions were: initial denaturalization of 94°C for 60 sec, and 36 cycles of 94°C for 60 sec, 55°C for 60 sec, 72°C for 2 min, and a final extension of 72°C for 8 min. The rDNA was PCR-amplified and sequenced twice for each organism, in order to screen for intragenomic polymorphisms (IGP). The first time the entire region (ITS1-5.8S- ITS2) was amplified and directly sequenced. In a second set of reactions we used internal primers (ITS2 5′-GCTGCGTTCTTCATCGATGC-3′, ITS3 5′-GCA TCG ATG AAG AAC GCA GC-3′) to amplify and sequence the internal transcribed spacers separately: ITS1 (using primers ITS5 and ITS2) and ITS2 (using primers ITS3 and ITS4).

PCR products were purified using ExoSAP-IT® (USB, Cleveland, OH), and sequenced using BigDye Terminator version 3.1 following manufacturer protocols. Products were analyzed in an ABI PRISM™ 377 DNA Sequencer (Applied Biosystems, Foster City, CA). *Aplysina*-sequences were deposited in GenBank (Table 2).

#### Sequence analyses

Sequences were verified and edited with Codon Code Aligner 2.0.1 (CodonCode Corporation). BLAST (NCBI/Blast) searches were used to verify the identity of sequences and check for possible contamination. Phylogenetic analyses included previously published rDNA sequences of *Aplysina* from the Caribbean and sequences of *Aiolochroia crassa* and *Verongula gigantea*, the latter were used as outgroup (Table 2). We reconstructed a Neighbor-Joining (NJ) tree, using a matrix of maximum composite likelihood distances, and a maximum parsimony (MP) tree, using an exact branch-and-bound search, as implemented in Mega 5.04 [82]. For these, non-parametric bootstrap (10,000 pseudo-replicates) was used to assess branch support. In addition, a Bayesian inference analysis was performed with MrBayes 3.1.2 [83] using the HKY model of sequence evolution as obtained with jModelTest 0.1 [84]. The program was run with four Markov chains (one cold and three heated) each 1,000,000-generation long, which were sampled every 100th trees and a burn-in of 2,500. Posterior probabilities were computed from the remaining trees.

### Nomenclatural Acts

The electronic version of this document does not represent a published work according to the International Code of Zoological Nomenclature (ICZN), and hence the nomenclatural acts contained in the electronic version are not available under that Code from the electronic edition. Therefore, a separate edition of this document was produced by a method that assures numerous identical and durable copies, and those copies were simultaneously obtainable (from the publication date noted on the first page of this article) for the purpose of providing a public and permanent scientific record, in accordance with Article 8.1 of the Code. The separate print-only edition is available on request from PLoS by sending a request to PLoS ONE, 1160 Battery Street, Suite 100, San Francisco, CA 94111, USA along with a check for $10 (to cover printing and postage) payable to “Public Library of Science”.

In addition, this published work and the nomenclatural acts it contains have been registered in ZooBank, the proposed online registration system for the ICZN. The ZooBank LSIDs (Life Science Identifiers) can be resolved and the associated information viewed through any standard web browser by appending the LSID to the prefix “http://zoobank.org/”. The LSID for this publication is: urn:lsid:zoobank.org:pub:96218730-8498-4814-8D9B-A371A678093B.
